# Temporal distribution characteristics of *Salmonella* foodborne disease in China based on the circular distribution method

**DOI:** 10.3389/fpubh.2026.1749011

**Published:** 2026-04-10

**Authors:** Wenli Diao, Yunchang Guo, Xiangyun Liu, Yiming Pei, Xinling Yu, Kailin Wang, Chengxin Ma, Jikai Liu

**Affiliations:** 1 Liaoning Center for Disease Prevention and Control, Shenyang, Liaoning, China; 2 Institute of Preventive Medicine, China Medical University, Shenyang, Liaoning, China; 3 NHC Key Laboratory of Food Safety Risk Assessment, Food Safety Research Unit (2019RU014) of Chinese Academy of Medical Science, China National Center for Food Safety Risk Assessment, Beijing, China; 4 China Medical University, Shenyang, Liaoning, China

**Keywords:** circular distribution method, foodborne disease, *Salmonella*, seasonal pattern, temporal distribution

## Abstract

**Objective:**

The temporal distribution characteristics of *Salmonella*-associated foodborne disease cases in China from 2014 to 2024 were examined to estimate the peak time point and epidemic period and to inform seasonal prevention efforts.

**Methods:**

Data on *Salmonella*-positive foodborne disease cases reported to the National Foodborne Disease Active Surveillance System during 2014–2024 were extracted. Circular distribution analysis was used to assess seasonal concentration and estimate peak timing, and Rayleigh’s Z test was used to evaluate seasonal clustering.

**Results:**

*Salmonella*-associated foodborne disease showed clear seasonality, with a mean peak time point occurring on July 17 and a peak epidemic period spanning from May 17 to September 26.

**Conclusion:**

*Salmonella*-associated foodborne disease in China shows a clear seasonal concentration, with a peak occurring in summer. These findings support prioritizing enhanced food safety supervision, targeted inspections of high-risk food settings, and strengthened public risk communication from May to September to reduce preventable exposures during the identified high-risk period.

## 1 Introduction

The circular distribution method can accurately determine the peak time point and the epidemic period of diseases and has been widely used to analyze the time distribution characteristics of various diseases, such as hand-foot-mouth disease, brucellosis, and other infectious diseases (1–4). It has also been applied to analyze seasonal variations in conditions such as preterm birth and mental disorders (5, 6), as well as to analyze the peak time of food poisoning or outbreaks (7, 8). However, the application of this method for identifying the peak period of foodborne diseases remains limited. In this study, we collected information on *Salmonella*-associated foodborne disease cases in China from 2014 to 2024, analyzed the time distribution characteristics, and obtained the peak time and epidemic period to provide a scientific basis for further prevention of foodborne diseases.

Previous studies on *Salmonella* and other foodborne pathogens have primarily described seasonality using monthly distributions or conventional time-series analyses. However, these approaches provide limited information on the precise timing and concentration of peak risk within a yearly cycle. Given that disease occurrence follows a natural annual periodic pattern, circular distribution analysis treats calendar time as cyclical data and enables estimation of both the average peak timing and the degree of seasonal concentration within a unified framework (9, 10). This approach may therefore provide clear evidence to identify high-risk periods and support targeted seasonal prevention strategies.

## 2 Information and methods

### 2.1 Sources of information

The case data used in this study were obtained from the National Foodborne Disease Active Surveillance System in China. This surveillance system is coordinated by the China National Center for Food Safety Risk Assessment (CFSA). Data from 2014 to 2024 were collected from active surveillance hospitals and analyzed, and cases identified positive for *Salmonella* infections using laboratory testing were defined as outpatient intestinal cases with diarrhea as the primary symptom, characterized by food or suspected to be caused by food, with three or more bowel movements per day, and with abnormal fecal characteristics such as loose stools, watery stools, mucus stools, or pus and blood stools. Case definitions, sampling procedures, and laboratory testing protocols followed the Foodborne Disease Surveillance Manual issued by CFSA (11). Biological samples were collected from surveillance cases and tested for *Salmonella* infections.

### 2.2 Method

#### 2.2.1 Collection of cases

Outpatient doctors in active surveillance hospitals collected and reported basic demographic information for patients who met the surveillance case definition. Special personnel were assigned to each active surveillance hospital to review the data, and the data were reviewed by CDCs at all levels.

#### 2.2.2 Laboratory test

The detection and serotyping of *Salmonella* were performed at each active surveillance hospital in accordance with the methods specified in the “Foodborne Disease Surveillance Manual” issued by the National Center for Food Safety Risk Assessment. Laboratory identification and serotyping of *Salmonella* were conducted in accordance with the standardized procedures described in the Foodborne Disease Surveillance Manual.

### 2.3 Statistical analysis

In this study, Microsoft Excel 2010 was used to organize the data, and R v4.2.0 was used to characterize the temporal distribution of *Salmonella* foodborne diseases and their peak characteristics were analyzed in China from 2014 to 2024, and the circular distribution method was used to statistically test whether there was a concentration trend in the occurrence of cases and their peak characteristics. The Mann–Kendall trend test was used to analyze the temporal trend of detection rates. The Mann–Kendall trend test is a non-parametric method widely used to detect monotonic trends in environmental and epidemiological time-series data (12). It was selected because it does not require normally distributed data and is robust for detecting the monotonic trends in surveillance time series. The circular distribution analysis is a statistical method that transforms data with periodic change characteristics into angles for analysis through the substitution principle of trigonometric functions to find out the direction of concentration of morbidity, the degree of dispersion, and the peak period of disease prevalence. A *p*-value of < 0.05 indicates that the difference is statistically significant.

### 2.4 Circular distribution method

The average angle of the circular distribution indicates the concentration direction of the onset time. The number of cases is counted by month, the onset time is converted into an angle, and then, through the principle of trigonometric substitution, the concentration direction of disease, the degree of dispersion, and the peak time of disease were estimated. Circular distribution analysis is a commonly used method in circular statistics for analyzing periodic or seasonal data and has been widely applied in epidemiological studies (9). The specific method is as follows: If 365 days are evenly divided into 360 degrees, 1 day is equivalent to 0.9863 degrees. The monthly median value was used as the group median value and converted to an angle, that is, 15.78 degrees in January, 44.87 degrees in February, and so on. Table 1 shows the angle conversion values for each month. Based on the principle of trigonometric substitution for the average angle “a” and the angle of the standard deviation “s,” and the number of *Salmonella*-positive cases at the peak time of the epidemic was calculated (a ± s).


The formula isasfollows:X=∑(ficosai)∕∑fi

$$Y=\sum (\mathrm{fi}\;\sin\;\mathrm{ai})∕\sum \mathrm{fi}\;R=\surd x^{2}+Y^{2}$$

$$\sin\;a=Y/R\;\cos\;a=X/R\;s=122.9548\surd \mathrm{lgR}\;Z=\sum {\mathrm{fi}}^{*}R^{2}$$

**Table 1:** Angle conversion values for each month.

Months	Range of days per month/d	Mean angle (a)	Radian (ai)
1	1~31	15.7808	0.2754
2	32~59	44.8767	0.7832
3	60~90	73.9726	1.2911
4	91~120	104.0574	1.8161
5	121~151	134.1370	2.3411
6	152~181	164.2192	2.8662
7	182~212	194.3014	3.3912
8	213~243	224.8767	3.9248
9	244~273	254.9589	4.4499
10	274~304	285.0410	4.9749
11	305~334	315.1233	5.4999
12	335~365	345.2055	6.0250
total			

Note: fi indicates the number of patients reported each month (i = 1, 2, ……0.12), ai indicates the angle of each month, R is an indicator of angular dispersion, which indicates the centralized trend of the circular distribution, a indicates the mean angle, and s indicates the standard deviation of the angle. Rayleigh’s Z test was used, and Z > Z0.05 was considered statistically significant.

In circular distribution analysis, the mean angle (a) represents the average timing of disease occurrence within a year and can be converted to a calendar date. The mean resultant length (R) reflects the degree of seasonal concentration, with larger values indicating stronger clustering around the mean peak time. The circular standard deviation (s) describes the dispersion of cases across the annual cycle. Rayleigh’s Z test is used to assess whether the observed seasonal pattern differs significantly from a uniform distribution. Rayleigh’s Z test was chosen because it is a standard test in circular statistics for assessing whether events are uniformly distributed over the year or show significant seasonal clustering.

## 3 Results

### 3.1 Detections of *Salmonella*-associated foodborne disease

A total of 1,409,123 individuals were tested for *Salmonella* at national foodborne disease active surveillance hospitals from 2014 to 2024, and 80,414 positive cases were detected, resulting in a detection rate of 5.71%. After the Mann–Kendall trend test, Z = 3.737 > 0. The results showed that the positive detection rate increased with time (*p* = 0.00018 < 0.01), rejecting the null hypothesis and indicating a significant upward trend. The linear regression model was Y = 0.4952X + 2.642. 2024 reported the highest number of positive cases (Table 2).

**Table 2:** Number and composition ratio of foodborne disease cases due to *Salmonella* infections in China from 2014 to 2024.

Year	Number of cases tested	Number of *Salmonella*-positive cases	Positive detection rate(%)	Composition ratio of positive cases(%)
2014	90,475	2,852	3.15	3.55
2015	110,564	3,640	3.29	4.69
2016	121,371	4,912	4.05	6.33
2017	113,406	5,436	4.79	7.01
2018	116,545	6,419	5.51	8.28
2019	124,872	7,553	6.05	9.74
2020	124,402	8,455	6.80	10.90
2021	144,836	10,007	6.91	12.90
2022	128,650	8,657	6.73	11.16
2023	159,483	10,641	6.67	13.72
2024	174,519	11,842	6.79	15.27
Total	1,409,123	80,414	5.71	100.00

### 3.2 Detection time distribution of *Salmonella*-associated foodborne diseases

**Figure 1:**
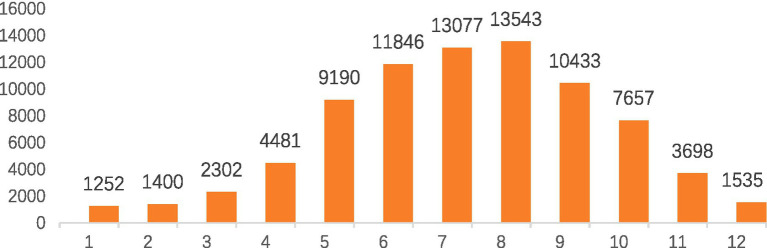
Monthly distribution of *Salmonella*-positive cases reported by national foodborne disease active surveillance hospitals in China from 2014 to 2024. The figure shows the aggregated number of laboratory-confirmed *Salmonella* cases by month, illustrating the seasonal pattern of disease occurrence.

The reported positive cases were concentrated between May and September, accounting for 72.24% (58,089/80414) of the total number of cases. Monthly case counts increased from May and remained elevated through September, consistent with the estimated epidemic period, as shown in Figure 1.

### 3.3 Peak incidence of *Salmonella*-positive foodborne disease

The circular distribution method was used to test whether there was a centralized trend in the distribution of *Salmonella*-positive cases reported by foodborne disease active surveillance hospitals. The results showed that there was a centralized trend in the distribution of *Salmonella*-positive cases reported by foodborne disease active surveillance hospitals across the country during 2014–2024. The peak time was July 17th, and the peak time zone was from May 17th to September 26th.

According to the formula in the statistics step, X = -0.4345, Y = -0.1934, R = 0.4756, sin a = −0.2558, cos a = −0.9136, and Z = 18189.2737. Based on the R value, checking the circular distribution bounding table, R > R0.05. Therefore, the null hypothesis was rejected, and the difference in the mean angle a was statistically significant, indicating a significant concentration trend in the distribution of *Salmonella*-positive cases. The results of Rayleigh’s Z test showed that Z = 18189.2737 > Z0.05 = 2.9957, indicating statistical significance and the presence of a mean angle.

Based on the negative values of both sin a and cos a, it was deduced that a was in the third quadrant, a = 195.6422465°, which was converted to a date by the angle, and the corresponding date was July 17th, which was the peak time point of *Salmonella*-positive cases reported by foodborne disease active surveillance hospitals in China. According to s = 69.8528, the peak time zone of *Salmonella*-positive cases was 195.6422465 ± 69.8528, and the corresponding date range was from May 17th to September 26th. The specific calculation process is shown in Table 3.

**Table 3:** Average angle calculation of *Salmonella*-positive cases reported by national foodborne disease active surveillance hospitals from 2014 to 2024.

Months	Mean angle(a)	Radian (ai)	Monthly cases (fi)	sin ai	cos ai	fi sin ai	fi cos ai
1	15.7808	0.2754	1,252	0.2720	0.9623	340.544	1204.7996
2	44.8767	0.7832	1,400	0.7056	0.7086	987.84	992.04
3	73.9726	1.2911	2,302	0.9611	0.2761	2212.4522	635.5822
4	104.0574	1.8161	4,481	0.9701	−0.2429	4347.0181	−1088.4349
5	134.1370	2.3411	9,190	0.7177	−0.6964	6595.663	−6399.916
6	164.2192	2.8662	11,846	0.2720	−0.9623	3222.112	−111399.4058
7	194.3014	3.3912	13,077	−0.2470	−0.9690	−3230.019	−12671.613
8	224.8767	3.9248	13,543	−0.7056	−0.7086	−9555.9408	−9596.5698
9	254.9589	4.4499	10,433	−0.9657	−0.2595	−10075.1481	−2707.3635
10	285.0410	4.9749	7,657	−0.9657	0.2595	−7394.3649	1986.9915
11	315.1233	5.4999	3,698	−0.7056	0.7086	−2609.3088	2620.4028
12	345.2055	6.0250	1,535	−0.2554	0.9668	−392.039	1484.038
Total			80,414			−15551.1913	−34939.4489

### 3.4 Peak *Salmonella-associated* foodborne illnesses by year, 2014–2024

The circular distribution method was used to calculate and test the peak time points and peak time zones for *Salmonella*-positive cases reported by national foodborne disease active surveillance hospitals from 2014 to 2024. There was a trend of a centralized distribution of reported cases in all years (*p* < 0.05), and the peak time point was in July. The peak time zone in 2018 and 2023 was from May to September, whereas in other years it extended from May to October, with no clear trend. The specific results are shown in Table 4.

**Table 4:** Peak time zone of *Salmonella*-positive cases reported by national foodborne disease active surveillance hospitals from 2014 to 2024.

Year	R	Z	P	a ± s	Peak time point	Lower limit of the peak time zone	Upper limit of peak time zone
2014	0.4985	626.2963	<0.05	207.3231 ± 67.6039	July 29th	May 21st	October 5th
2015	0.4356	636.424	<0.05	206.2268 ± 73.8659	July 28th	May 14th	October 11th
2016	0.4718	1011.0114	<0.05	204.4141 ± 70.2241	July 26th	May 16th	October 5th
2017	0.4693	1106.9835	<0.05	200.1163 ± 70.4758	July 22nd	May 11th	October 1st
2018	0.4946	1480.3873	<0.05	199.4745 ± 67.9888	July 21th	May 13th	September 28th
2019	0.4825	1667.1383	<0.05	204.152 ± 69.1724	July 26th	May 16th	October 4th
2020	0.4908	1955.6379	<0.05	204.8384 ± 68.3538	July 26th	May 16th	October 4th
2021	0.4652	2121.3626	<0.05	200.1271 ± 70.8808	July 22th	May 11th	October 2nd
2022	0.4834	1941.4096	<0.05	203.5974 ± 69.0866	July 25th	May 16th	October 3rd
2023	0.4459	2115.8244	<0.05	204.3420 ± 72.8191	July 26th	May 13th	September 28th
2024	0.4749	2898.0700	<0.05	209.6529 ± 69.920	July 30th	May 20th	October 9th

## 4 Discussion

In this study, the circular distribution method was used to analyze the occurrence of *Salmonella*-associated foodborne disease in China each month from 2014 to 2024 and to explore the seasonal characteristics of its occurrence. The results showed an obvious seasonal pattern of peaks and monthly concentrations of *Salmonella*-associated foodborne disease; the average peak incidence was on 17th July, and the peak epidemiological period ranged from 17th May to 26th September. The peak time of *Salmonella*-associated foodborne disease in this study occurred during summer, likely due to high temperatures, which is consistent with the seasonal distribution of general foodborne pathogen infections. The number of positive cases and detection rate were higher than those in other months, suggesting that the temperature during this period was suitable for the reproduction of *Salmonella* (12), which is consistent with the time period of the onset of the outbreaks or disseminated cases reported in the literature (13). In addition, public dietary habits, such as eating cold and raw food in summer, increases the chances of *Salmonella* infection. The peak time point and time zone of foodborne diseases caused by *Salmonella* fluctuated annually, which was consistent with the fluctuation of high temperatures. Therefore, temperature is closely related to the occurrence of foodborne diseases.

The observed upward trend in the positive detection rate of *Salmonella* over the study period should be interpreted with caution. Although this trend may indicate a true increase in disease burden, it could also be influenced by improvements in laboratory diagnostic capacity, increased healthcare-seeking behavior, changes in surveillance coverage, or enhanced reporting practices over time. Because this study is based on surveillance data, it was not possible to fully disentangle these factors. Future studies incorporating information on testing intensity, participating sites, and diagnostic practices would help clarify the drivers of the observed increase.

Previous studies have also demonstrated the correlation between seasons and the occurrence of foodborne diseases (14). Foodborne diseases are caused by bacteria, fungi, parasites, and viruses, and climate change alters the survival of many foodborne pathogens in nature. It also modifies transmission factors such as human behavior and wildlife vectors, which may alter the prevalence of illnesses (15–17). The United States study analyzed *Salmonella* surveillance data from the Foodborne Disease Active Surveillance Network (FoodNet) and national weather data and found that extreme heat increases the prevalence of several of the most prevalent *Salmonella* serotypes, such as *Salmonella enteritidis* and *Salmonella typhimurium* (18).

The circular distribution method was used to analyze the seasonal and monthly distribution of *Salmonella* foodborne diseases in China. According to the peak time of *Salmonella* foodborne diseases, effective prevention and control measures can be implemented early, and public education on preventing *Salmonella* foodborne diseases should be carried out to improve the food safety awareness of citizens. Considering the fecal–oral transmission of *Salmonella*, food hygiene supervision should be intensified in summer.

In addition, the identification of a clear seasonal peak period provides an opportunity for public health authorities to implement targeted and actionable interventions. These may include strengthening seasonal food safety inspections, enhancing early warning systems for foodborne disease outbreaks, and improving risk communication to the public during high-risk months, thereby reducing the burden of *Salmonella*-associated foodborne disease.

This study has several limitations. First, detailed demographic information such as age group, sex, and geographic region was not available for all cases, which limited stratified analyses of seasonal patterns. Second, information on *Salmonella* serotypes was not included in the present analysis, preventing the assessment of potential differences in seasonal characteristics among serotypes. Future studies incorporating demographic, geographic, and serotype-specific data would help to better identify high-risk populations and refine targeted prevention strategies.

Overall, our findings support the strengthening of food-safety interventions during the identified high-risk months.

## Data Availability

The datasets presented in this study can be found in online repositories. The names of the repository/repositories and accession number(s) can be found in the article/supplementary material.
